# Assessing extremely negative online patient reviews and complaints of musculoskeletal oncology surgeons in the United States: a retrospective analysis

**DOI:** 10.1186/s13018-024-04881-y

**Published:** 2024-07-23

**Authors:** Kyle J. Hitchman, Anthony N. Baumann, Sarah E. Welch, Albert T. Anastasio, Kempland C. Walley, William Eward

**Affiliations:** 1 Department of Emergency Medicine, University of Louisville, Louisville, KY USA; 2 College of Medicine, Northeast Ohio Medical University, Rootstown, OH USA; 3 Campbell University School of Osteopathic Medicine, Lillington, NC USA; 4 Department of Orthopaedics, Duke University, Durham, NC USA; 5 Department of Orthopaedics, University of Michigan, Ann Arbor, MI USA

**Keywords:** Physician review websites, Online reviews, Patient satisfaction, Consumer preference, Decision making preference

## Abstract

**Introduction:**

Physician-review websites (PRWs) are commonly used by patients while searching for a surgeon. There is no current literature investigating the factors that contribute to online one-star reviews of musculoskeletal oncology surgeons. This retrospective study aims to identify these factors to determine areas of care affecting patient’s subjective reviews.

**Methods:**

Patient ratings and comments regarding musculoskeletal oncology surgeons from the Musculoskeletal Tumor Society (MSTS) were collected from Vitals.com. One-star reviews with comments were then classified as either operative or nonoperative. These complaints were then further classified based on content including wait time, uncontrolled pain, time spent with the physician, surgical outcomes, medical staff/institutional complaints, and bedside manner.

**Results:**

A total of 169 reviews (375 complaints) from 181 physicians were included. Of these complaints, 198 were from patients in the operative category while 177 were from patients in the nonoperative category. Bedside manner was the most common complaint. Operative patients reported higher instances of uncontrolled pain in their reviews, whereas nonoperative patients more frequently cited wait time. No significant difference in the complaints that mentioned the amount of time spent with the physician, bedside manner, a disagreement with the plan, or the medical staff or institution was found.

**Conclusion:**

Online one-star reviews of musculoskeletal oncology surgeons on Vitals.com referenced both surgical and non-surgical aspects of patient encounters, with bedside manner being the most popular complaint overall. Surgical patients were more likely to complain of uncontrolled pain whereas non-operative patients were more likely to complain of wait time.

**Type of study:**

Outcomes 2c.

## Introduction

Physician-review websites (PRWs) continue to grow in popularity and impact among prospective patients who are searching for a physician [1]. A study conducted by Hanauer et al. [2] showed that 68% of patients admit to utilizing a PRW while searching for a new physician, regardless of specialty. Recent literature has shown that there is no correlation between physician mortality rate and their numerical rating on PRWs [3]. Interestingly, among physicians who scored in the lowest quartile of performance scores for their respective specialties, only a minority (5 − 32%) were found to be in the lowest quartile based on reviews found on PRWs [4], suggesting that there is a difference between relatively objective performance measures and subjective patient reviews. Despite this finding, 37% of patients who utilize PRWs said they would avoid a physician with a low numerical score and poor reviews [5]. Research has shown that physicians with low ratings on PRWs are more likely to score poorly in categories comprised on interpersonal skills, including communication and bedside manner [6, 7]. Despite the lack of a correlation between negative online reviews found on PRWs and related poor outcomes, the majority (78%) of physicians report an increase in work-related stress secondary to PRWs [8].

There are several studies that have characterized and examined the factors that contribute to negative reviews on PRWs within orthopaedic surgery, but, to the best of the authors’ knowledge, there has been no research focusing on the complaints of one-star reviews on PRWs for orthopaedic surgeons subspecializing in musculoskeletal oncology [9–13]. Previous literature examining the contributing factors of one-star reviews on PRWs currently exists for orthopaedic-trained surgeons within the subspecialties of trauma, arthroplasty, spine and sports medicine [9, 10, 12–16]. While many PRWs are available for patients to access, Vitals.com is a popular PRW and has been used in previous research characterizing negative online reviews of orthopaedic surgeons trained in other subspecialties [9, 10, 13]. The purpose of this study was to characterize the factors that contributed to one-star reviews of musculoskeletal oncology surgeons on Vitals.com to ascertain which factors lead to very low patient satisfaction.

## Methods

### Study set-up

This study was a retrospective analysis examining extremely negative online “one-star reviews” of musculoskeletal oncology surgeons in the United States as recorded on Vitals.com. Vitals.com is an online platform where healthcare professional can be found by searching and filtering by specialty, geographic region, name, or several other factors. Vitals.com allows users to write reviews regarding their experiences with healthcare providers that can be read by future website users to aid in the process of choosing the best physician for them based on what is found in the reviews. These reviews are written on a scale of one to five stars, with five stars being the highest rating a reviewer can give their provider. This study was completed in July 2023 and involved all Vitals.com records from inception of the database until July 2023. This study examined all of the physicians in the United States that were part of the Musculoskeletal Tumor Society (MSTS), a prominent organization for musculoskeletal oncology surgeons, that could be found on Vitals.com.

### Eligibility criteria

Inclusion criteria were orthopedic surgeons from the United States who were members of MSTS who could be found on Vitals.com when searching their name. Each physician found on the MSTS website was verified to be an oncology orthopedic surgeon via an independent internet search. Exclusion criteria was physicians who did not practice in the United States, were not members of MSTS, and could not be found on Vitals.com. The entire publicly available directory for MSTS was searched on Vitals.com. Physicians were also excluded if the data was clearly incorrect on Vitals.com or unable to be retrieved on Vitals.com due to a website error.

### Data and category definitions

For the purposes of this study, a “review” represents a star rating on Vitals.com, from one-star to five-star, with one-star reviews representing extremely negative reviews. A “comment” refers to the written material provided by the patient along with the review and can be either positive, negative, or neutral. However, for the purposes of this study, a “complaint” refers to a negative comment that accompanies a one-star review, representing a negative expression from the patient. As a complaint is the written text of the patient, multiple complaints could exist on a single one-star review. This study stratified patient complaints into the “Operative” group or the “Non-Operative” group. Patient complaints in the Operative group represented patients who received any form of surgical intervention at any time by the physician. In contrast, patient complaints in the Non-Operative group represented patients who did not receive any form of surgical intervention at any time by the physician as well as patient complaints that could not be classified due to vague descriptions (e.g., “the experience was terrible”). The distinction between the two groups was made based on content of the written review. Operative reviews were those in which the review suggested some sort of procedure or surgery had been performed. Nonoperative reviews were those that failed to mention that a procedure had been performed. After stratification into groups based on operative status, complaints were further sub-stratified into categories: not enough time spent with the provider, wait time, bedside manner and patient experience, surgical complications or outcomes, disagreement with decision or plan, uncontrolled pain, and complaints related to medical staff or institution. For the purpose of classifying complaints in this study, “not enough time spent with the provider” indicated any complaints related to limited face-to-face interactions with the physicians, short visit times, or other similar complaints. The category of “wait time” indicated any complaints related to long wait times, delay in seeing the physician, inability to get an appointment due to long wait times or being seen by the physician significantly later than scheduled. The category of “bedside manner and patient experience” indicates any complaint related to the surgeon’s attitude, personality, demeanor, relationship, compassion, or any general non-descriptive patient emotion or expression related to the physician visit (e.g., “this was a terrible experience” or “he was incredibly mean”). The category of “surgical complications or outcomes” indicates any complaints related to the outcome, result, or subsequent complication due to a surgical procedure, excluding uncontrolled pain, which was placed into a separate category. The category of “disagreement with decision or plan” indicates complaints related to when a patient did not like or agree with the physician’s diagnosis, plan of care, or medical decision-making. The category of “uncontrolled pain” indicates complaints related to pain levels. The category of “medical staff or institutional complaints” indicates complaints related to the medical staff other than the surgeon or complaints related to the institutional workflow or policies.

### Data collection

Data collection was performed by multiple authors. Data collected included the number of surgeons from the MSTS directory who could be found on Vitals.com, the total number of reviews, the number of one-star reviews, the number of complaints, category of patient type (Operative group or Non-Operative group), and the number of complaints in each of the included complaint categories.

### Statistical analysis

This study utilized the Statistical Package for the Social Sciences (SPSS) version 29.0 (Armonk, NY: IBM Corp) for statistical analysis. Frequency counts and descriptive statistics were used to describe the data as needed. Binary comparisons between groups were completed using the Chi-square test. Statistical significance was set at 0.05.

## Results

### Search results

A total of 186 physicians were initially included in this study; however, five physicians were excluded due to clearly incorrect information (e.g., extremely positive comments with a one star-review) or errors on the Vitals.com website (e.g., no information provided). From the 181 included physicians, there were a total of 1,526 total reviews (one-star through five-star) with comments. From those 1,526 reviews with comments, 169 reviews (11.1%) represented one-star reviews with 72 one-star reviews (42.6%) in the Operative group and 97 one-star reviews (57.4%) in the Non-Operative group. From the 169 one-star reviews, a total of 375 complaints were assessed, representing an average of 2.2 complaints per one-star review (Table 1).

**Table 1:** Demographic data for this study. Data includes the number of physicians included, total reviews, total one-star reviews, one-star reviews in both groups, total complaints, complaints in both groups, and the average number of complaints per one-star review

Categories	Values
Number of physicians included, n	181
Total reviews, n (%)	1526 (100%)
Total one star reviews, n (%)	169 (11.1%)
One-star reviews in the Operative group, n	72
One-star reviews in the Non-Operative group, n	97
Total complaints, n (%)	375 (100%)
Complaints in the Operative group, n (%)	198 (52.8%)
Complaints in the Non-Operative group, n (%)	177 (47.2%)
Average number of complaints per one-star review	2.2

### Classification of one-star complaints

From the 375 complaints assessed in this study from 169 one-star reviews of musculoskeletal oncology surgeons on Vitals.com, 198 complaints (52.8%) were in the Operative group and 177 complaints (47.2%) were in the Non-Operative group. There were 35 complaints (9.3% of total complaints) due to not enough time spent with the physician with 14 complaints (7.1% of group complaints) in the Operative group and 21 complaints (11.9% of group complaints) in the Non-Operative group. There were 34 complaints (9.1% of total complaints) due to wait time with 7 complaints (3.5% of group complaints) in the Operative group and 27 complaints (15.3% of group complaints) in the Non-Operative group. There were 132 complaints (35.2% of total complaints) due to bedside manner and patient experience with 59 complaints (29.8% of group complaints) in the Operative group and 73 complaints (41.2% of group complaints) in the Non-Operative group. There were 54 complaints (27.3% of group complaints) due to surgical complications or outcomes in the Operative group. There were 58 complaints (15.5% of total complaints) due to disagreement with the physician decision or plan with 28 complaints (14.1% of group complaints) in the Operative group and 30 complaints (16.9% of group complaints) in the Non-Operative group. There were 28 complaints (7.5% of total complaints) due to uncontrolled pain with 24 complaints (12.1% of group complaints) in the Operative group and 4 complaints (2.3% of group complaints) in the Non-Operative group. There were 34 complaints (9.1% of total complaints) due to medical staff or institutional complaints with 12 complaints (6.1% of group complaints) in the Operative group and 22 complaints (12.4% of group complaints) in the Non-Operative group. Overall, the most common complaint was due to physician bedside manner and patient experience with 35.2% of total complaints. The two most common complaints in the Operative group were bedside manner and patient experience (29.8% of group complaints) and surgical complications or outcomes (27.3%). The two most common complaints in the Non-Operative group were bedside manner and patient experience (41.2% of group complaints) and disagreement with the physician’s decision or plan (16.9% of group complaints) (Table 2).

**Table 2:** Type of complaint in total and per group by category

One-star complaint category	Operative group complaints (% of operative group)	Non-operative complaints (% of non-operative group)	Total complaints (%)
Not enough time with physician	14 (7.1%)	21 (11.9%)	35 (9.3%)
Wait time	7 (3.5%)	27 (15.3%)	34 (9.1%)
Bedside manner and patient experience	59 (29.8%)	73 (41.2%)	132 (35.2%)
Surgical outcomes or complications	54 (27.3%)	-	54 (14.4%)
Disagree with the physician decision or plan	28 (14.1%)	30 (16.9%)	58 (15.5%)
Uncontrolled pain	24 (12.1%)	4 (2.3%)	28 (7.5%)
Medical staff or institution	12 (6.1%)	22 (12.4%)	34 (9.1%)
Total number of complaints	198 (100%)	177 (100%)	375 (100%)

### Complaint comparisons between the operative and non-operative groups

**Fig. 1:**
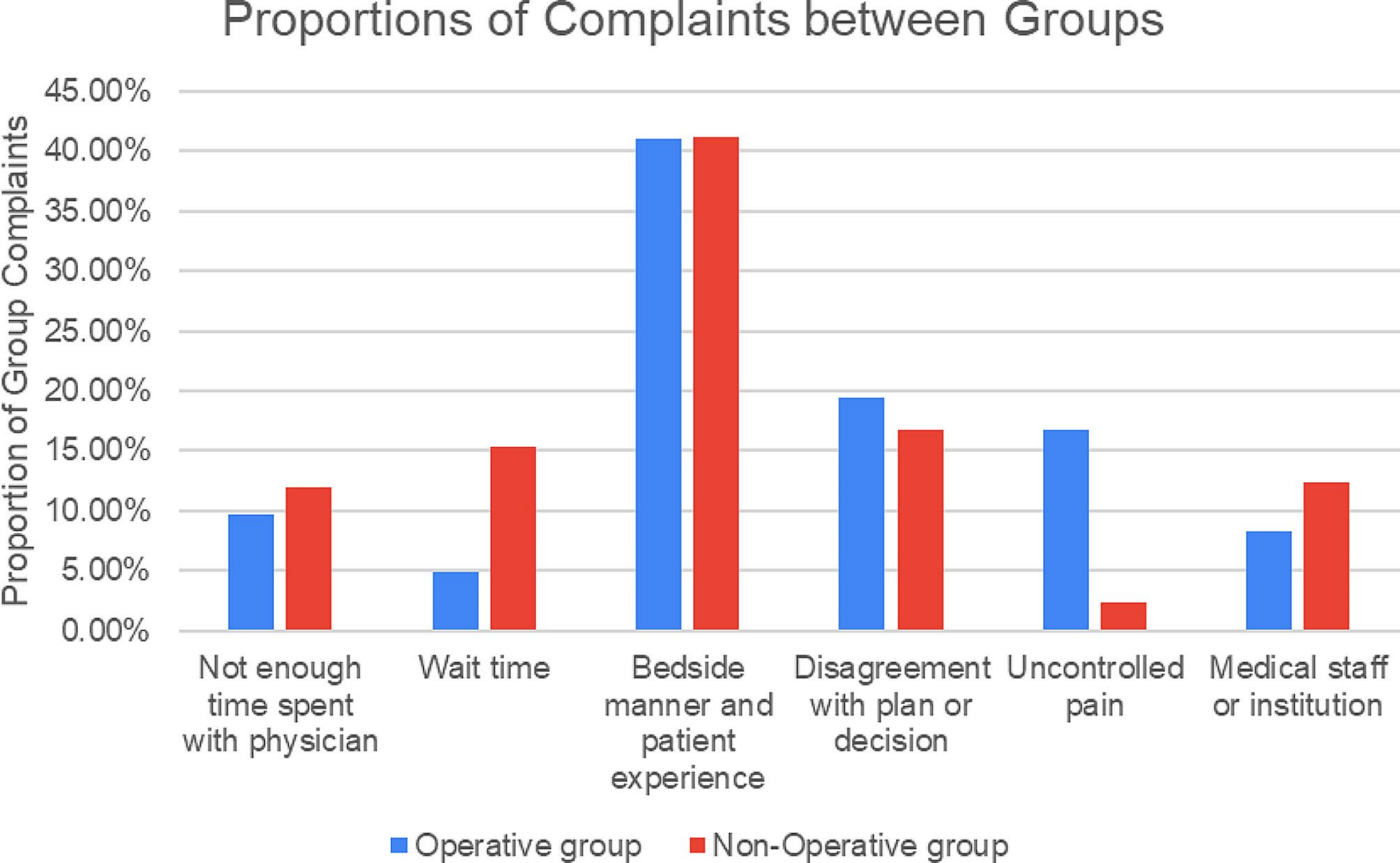
Proportions of complaints per group for the Operative group and the Non-Operative group. Categories of complaints included not enough time spent with the physician, wait time, bedside manner and patient experience, disagreement with plan or decision, uncontrolled pain, and medical staff or institution. For direct comparison purposes, the surgical outcomes or complications category was left out as this category only applied to the Operative group and not the Non-Operative group

This study also examined the relative proportions of each complaint between the Operative and Non-Operative groups, excluding complications related to surgical complications or outcomes in order to allow for a fair comparison between groups. There was no significance difference between the proportion of complaints related to not enough time spent with the physician in the Operative group compared to the Non-operative group (9.7% versus 11.9%; *p* = 0.540). However, there was a significant difference in the proportion of complaints related to wait time between the two groups with the Non-Operative group having a significantly greater proportion of complaints as compared to the Operative group (15.3% versus 4.9%; *p* = 0.003). There was no significant difference between the proportion of complaints related to bedside manner and patient experience between the Operative group and the Non-Operative group (41.0% versus 41.2%; *p* = 0.961). Furthermore, there was no significant difference between the proportion of complaints related to disagreement with the physician’s decision or plan between the Operative group and the Non-Operative group (19.4% versus 16.7%; *p* = 0.563). However, there was a significant difference in the proportion of complaints related to uncontrolled pain between the two groups with the Operative group having a higher proportion as compared to the Non-Operative group (16.7% versus 2.3%; *p* < 0.001). Finally, there was no significant difference in the proportion of complaints related to medical staff or the surgeon’s institution in the Operative group compared to the Non-Operative group (8.3% versus 12.4%; *p* = 0.236). Overall, the Non-Operative group had a significantly higher proportion of complaints related to wait time whereas the Operative group had a significantly higher proportion related to uncontrolled pain (Fig. 1).

## Discussion

Patients are relying on PRWs during their initial search for a potential new physician more than ever before, which aligns with the continuing shift towards a consumeristic focus in the health care market [2, 17]. Considering the fact that at least one-third of patients will avoid a physician with more negative reviews, it is crucial that physicians are aware of and understand the factors that go into these one-star reviews on PRWs [3, 5]. The results of this study indicate that one-star reviews of musculoskeletal oncologists found on Vitals.com were written by patients who underwent surgical intervention as well as those who did not. While two categories of complaint – uncontrolled pain and wait times – reflected the nature of the care, the remaining types of complaints were consistent whether the interaction were operative or nonoperative. For example, it is not surprising that one-star complaints in the Operative category were significantly more likely to mention uncontrolled pain when compared to one-star comments written by patients who were not offered a surgical intervention. Nor is it surprising that patients who did not undergo surgical intervention were significantly more likely to reference a long wait time in their online review compared to patients in the operative group. Interestingly, these findings contradict previous research examining one-star reviews of orthopaedic surgeons trained in various other subspecialties, which tend to show that patients in the nonoperative group are more likely write negative reviews on PRWs [9, 13–15]. This may reflect the fact that most other orthopaedic subspecialties are relatively more elective than musculoskeletal oncology and have a greater number of practitioners from which patients can choose prior to committing to surgery.

Research has shown that physicians are reporting higher job-related stress coinciding with the increasing popularity of PRWs [1, 8]. However, it is important to note that a poor online rating does not correlate with lesser aptitudes of the respective physicians [3, 4, 16]. In a study of the factors contributing to negative one-star reviews of orthopaedic-trained arthroplasty surgeons on PRWs, it was found that most complaints did not mention the surgeon’s capabilities but rather their interpersonal skills [16]. Indeed, this reflects the fact that PRWs indicate patients’ *perceptions* of a surgeon’s abilities (whether that be the ability to communicate or the ability to operate). The findings of this study support this conclusion and show that most one-star reviews of musculoskeletal oncology surgeons found on Vitals.com mention a prolonged wait time. This is an interesting example because a prolonged wait time can often be the result of factors which are actually of benefit to patients – for example, a physician who allows patients in need to be overbooked or a physician who spends ample time with those patients who need it. It was not possible to determine if physicians receiving one-star reviews due to long wait times were less likely to receive complaints about amount of time spent with patients.

While research has proven that patients will avoid physicians with more one-star reviews on PRWs, it is important to note that the majority of reviews found on PRWs are overall positive in nature [5, 14]. In fact, a study conducted by Pollock et al. determined that more than 90% of reviews on PRWs are positive [14]. Our research suggested a similar trend regarding the reviews of musculoskeletal oncology surgeons on PRWs with only 11.1% of reviews receiving one-star. The impact of poor online reviews may be contributing to the increasing levels of job-associated stress reported by physicians, however, research has shown that physicians who strive to improve the non-clinical aspects of their practice, including bedside manner, shorter wait times, and increasing the amount of time spent in the room with a patient, can increase their overall rating on PRWs [8, 11, 12, 14, 18, 19].

This study is not free of limitations that may impact the interpretation of the data in this study. Vitals.com is only one of a vast network of PRWs that exist for patients to utilize, and this study solely characterized reviews found on Vitals.com. Despite its popularity as a PRW, it does allow patients to write anonymous reviews, which may lead to reviews being published for ulterior motives outside of a negative experience with a physician. Additionally, there were physicians found on the MSTS database that did not have their Vitals.com profiles set up appropriately or with enough detail to be included in the study. It remains unclear whether or not this is secondary to a coding issue with Vitals.com but clicking on some physician profiles resulted in an error message. Given the subjective nature of the data collection process in this study, there is the possibility of misclassification bias for the reviews characterized by this study. For example, if a patient did receive operative care, but did not mention this in their review, the review would have been incorrectly stratified into the nonoperative group. Similarly, if a patient did not receive operative care, but worded their review in a way that could be misconstrued by a reader to imply that they did in fact receive operative care, this review could have also been miscategorized during data collection of this study. Due to this, the number of complaints attributed to patients in the nonoperative cohort may have been falsely inflated. Finally, this study only included musculoskeletal oncology surgeons in the United States that were both listed in the MSTS database and had a profile on Vitals.com. This does reduce the generalizability of the study and prevents the findings of this study from being applied to other countries outside of the United States. More research is needed to determine if countries that are less susceptible to commercial influence have similar grievances with their physicians.

## Conclusion

In summary, this study found that negative one-star reviews of musculoskeletal oncology surgeons found on Vitals.com were equally written by patients who underwent surgical procedures as well as patients who only underwent nonoperative care. Poor bedside manner was the most common complaint regarding all of the included physicians. Patients who underwent surgery had a higher proportion of complaints related to controlled pain whereas patients who did not undergo surgery had a higher proportion of complaints related to wait time. Further research should be done to further analyze the impact of these one-star reviews on job-associated stress and overall job satisfaction in musculoskeletal oncology surgeons as well as overall patient care.

## Data Availability

No datasets were generated or analysed during the current study.
